# The Special Role of Higher-Frequency Neighbors at the Phonological Level: An Event-Related Potential Study of Chinese Character Naming

**DOI:** 10.1155/2013/579216

**Published:** 2013-07-02

**Authors:** Jing Zhao, John X. Zhang, Hong-Yan Bi

**Affiliations:** ^1^Key Laboratory of Behavioral Science, Institute of Psychology, Chinese Academy of Sciences, 16 Lincui Road, Chaoyang District, Beijing 100101, China; ^2^Graduate University of Chinese Academy of Sciences, Beijing 100049, China; ^3^Centre for Psychology Studies, Fudan University, Shanghai 200433, China

## Abstract

The present study explored the time course of neighborhood frequency effect at the early processing stages, examining whether orthographic neighbors with higher frequency exerted an influence on target processing especially at the phonological stage by using the event-related potential (ERP). Thirteen undergraduate students were recruited in this study, and they were required to covertly name Chinese characters with or without higher-frequency neighbors (HFNs); meanwhile, their brain activity was recorded. Results showed that the effect of neighborhood frequency was significant in frontocentral P2 amplitude, with a reduction for naming characters with HFNs compared to those without HFNs; while there was no effect in posterior N1 amplitude. The only neighborhood frequency effect in P2 component suggested a special role for the HFNs in phonological access of  Chinese characters. The decrease in amplitude for naming with-HFN characters might be associated with the phonological interference of higher-frequency neighbors due to their different pronunciations from the target characters.

## 1. Introduction

Once a single word is presented, its orthographically similar words are also partially activated. Coltheart first introduced the concept of *orthographic neighborhood *of a target word, defined as all words of the same length that can be generated by changing just one letter while preserving letter positions [1]. For example, *cheap*, *chest*, *cleat*, and *wheat* are all neighbors of *cheat*. Grainger and his colleagues pointed out that the printed frequency of a word's orthographic neighbors played an important role in identification process of this target word, which is termed as *neighborhood frequency effect *[2]. The authors indicated that if the frequency of a target word was not the highest among its neighbors, those higher-frequency neighbors (HFNs) would compete with the target word and, consequently, slow down its processing. This inhibition was reported in several studies of lexical decision [2–8]. In naming tasks, no effect or a facilitatory trend of neighborhood frequency was observed [4, 9]. Grainger [4] gave an explanation to the absence of neighborhood interference based on the analogy theory of word naming [10, 11]. The pronunciations in alphabetic orthographic neighborhoods were of high consistency, a word usually sounded similar with its orthographic neighbors, and then the neighbors with higher frequency would provide support for the component phonology of the target word [4]. However, there is a close relationship between visual forms and pronunciations in alphabetic words, thus it is still needed to determine whether the HFNs affect the target naming during phonological processing or visual/orthographic analysis. As opposed to alphabetic languages, Chinese with a logographic writing system lacks the grapheme-to-phoneme correspondence (GPC) rules, in which visual words map onto speech sounds through an addressed way [12, 13]. Thus, the orthography and phonology could be distinguished within Chinese characters.

Considering different language characteristics, the definition of orthographic neighbors for alphabetic words is inapplicable for Chinese characters. However, about 85% of Chinese characters are semantic-phonetic compounds [14, 15] with the phonetic and semantic radicals informing to some extent about the character's pronunciation and meaning, respectively. By the medium of phonetic or semantic radicals, orthographic neighborhood of Chinese characters can be formed. For instance, characters *圾* (pronounced/ji1/, meaning *garbage*; the number here refers to Chinese tone; the same below), *汲* (/ji2/, *draw*), *极* (/ji2/, *unmitigated*), *笈* (/ji2/, *book*), *岌* (/ji2/, *danger*), *级* (/ji2/, *class*), *吸* (/xi1/, *absorb*), and *靸* (/sa3/, *shoes*) have the same phonetic radical *及* (ji2, and) and are considered as a phonetic-radical orthographic neighborhood. Moreover, because phonological consistency is much lower in Chinese orthographic neighborhoods [16, 17], the higher-frequency neighbors were expected to inhibit the target naming according to analogy theory of word naming, and this has been proved. Li et al. examined the neighborhood frequency effect in Chinese character naming, in which neighbors with the highest frequency sounded differently from target characters in the with-HFN condition [18]. Results showed a significantly interference effect of neighborhood frequency, and participants spent longer time and made more errors in with-HFN condition than in without-HFN condition. And their follow-up fMRI study found that naming with-HFN characters induced greater activations than naming without-HFN characters in bilateral inferior frontal gyrus (bi-IFG) which was related with the phonological competition and inhibition of extraneous phonological activation [19]. The above findings revealed that higher-frequency neighbors exerted an inhibitory influence on Chinese-character naming, and this inhibition might be linked to the phonological processing. But whether the HFN effect was special to the phonological level was still unclear because of the low temporal resolution of brain imaging, and the event-related potential (ERP) technique is undoubtedly a valuable way to dissociate the phonological processing from other stages (e.g., visual stage). 

Of particular interests to the present study are N1 and P2 components. The bilaterally posterior N1 is usually considered as a visual-orthographic component, with amplitude peak at around 130 ms to 170 ms [20–26]. The P2 component occurs around 200 ms after stimulus at centrofrontal sites and indexes lexical phonology in Chinese reading [27–29]. A previous study of neighborhood frequency effect using lexical decision found that English words with HFNs reliably induced larger amplitude than that without HFNs in the 180–240 ms time window, while there was no difference between the two conditions at earlier stages, revealing the possible phonological influence of HFNs [30]. However, to our knowledge, there were no ERP researches on neighborhood frequency effect in naming tasks. So, the present study intended to use ERPs to examine the mechanism of neighborhood frequency effect in Chinese-character naming. According to the analogy theory of word naming, it could be expected that the higher-frequency neighbors would only affect the target processing at phonological stage, exhibiting an effect in P2 component but not N1 component.

## 2. Methods

### 2.1. Participants

Thirteen undergraduate students (7 males) took part in this study. The mean age was 22 years old. All the participants were right-handed native Mandarin speakers with normal or corrected-to-normal vision. Written consent was obtained from each participant before the experiment.

### 2.2. Design and Materials

There were 48 characters as targets, 24 for with-HFN and without-HFN conditions, respectively (see Table 1). For the without-HFN characters, a target character had the highest frequency within its neighborhood. For the with-HFN characters, at least one of its neighbors was of 3 per million higher frequency referred to as a previous study [18]; meanwhile, the target and its neighbor(s) with the highest frequency sounded differently from each other. For example, *诞* (pronounced /dan4/, meaning birth, character frequency being 7.09 occurrences per million; the same below), *涎* (/xian2/, saliva, 0.74), *蜒* (/yan2/, wriggly, 0.85), and *筵* (/yan2/, feast, 0.65) form an orthographic neighborhood. The target character is *诞*, and its orthographic neighbors all have much lower character frequency, so this target is considered as a without-HFN character. *狄* (/di2/, barbarians, 5.41), *秋* (/qiu1/, autumn, 24.03), *伙* (/huo3/, partner, 28.72), and *钬* (/huo3/, holmium, 0.02) form another neighborhood, and the target character is *狄*. The neighbor *秋* has the highest frequency in this neighborhood, and the difference in frequency between *狄* (5.41) and *秋* (24.03) is greater than 3 per million; meanwhile the two characters sound different from each other, and thus the target *狄* is regarded as a with-HFN character. The mean number of HFNs in one neighborhood was 2 (range, 1 to 6; standard deviation = 1.47).

The targets were irregular with a low level of consistency. The irregular character indicates the character whose pronunciation is different from that of its phonetic radical; for example, *靸* (sa3, *shoes*) is an irregular character, because of its phonetic radical *及* (ji2, *and*) with a different pronunciation. The consistency value (*c*) can be calculated from the relative ratio of the number of orthographic neighbors with the same pronunciation (*n*) to the whole neighborhood size (*N*), *c* = *n*/*N* [31]. For example in the phonetic-radical neighborhood of *及* (ji2, and), its neighborhood size is eight, and there are six neighbors with the same pronunciation/ji/, *圾* (/ji1/, garbage), *汲* (/ji2/, draw), *极* (/ji2/, unmitigated), *笈* (/ji2/, book), *岌* (/ji2/, danger), and *级* (/ji2/, class), producing a consistency level of /ji/of 0.75 (*c* = 6/8). In the present study, the consistency of each target character was below 0.4. The target characters were all left-right structure with the phonetic radical on the right side. Moreover, the number of neighbors, character frequency, stroke, and consistency were balanced between conditions (Table 2). Character frequency information was collected from the Chinese Text Computing database (http://lingua.mtsu.edu/chinese-computing/), and the neighborhood was defined on the basis of Li and Kang's statistics [32]. None of the target characters shared the same phonetic radical.

### 2.3. Procedure

**Figure 1:**
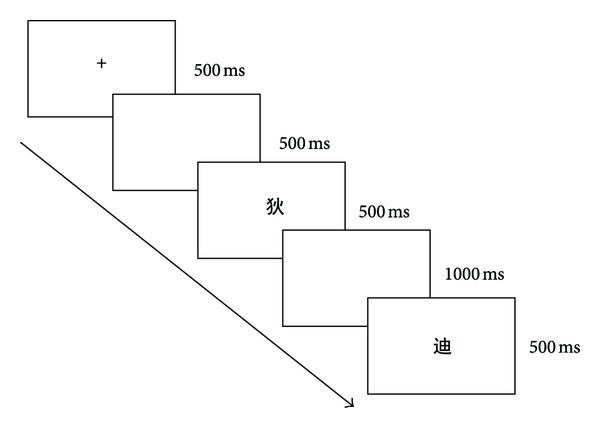
The presentation format of each trial.

Participants sat in a sound-attenuated room, at a viewing distance of 50 cm from the screen, with a visual angle of 5.7° × 5.7°. They were instructed to sit comfortably and concentrate on the stimulus avoiding head movements or any other unnecessary movements. Each participant was given 10 practice trials before the formal experiment.  Figure 1 displays the presentation format within each trial. A trial consisted of a 500 ms cross-fixation, a 500 ms blank screen, and a 500 ms target character. The participants were asked to silently name the target. To ensure that the phonology of the target character was accessed, the covert naming was followed with a homophonic decision, in which participants should make a decision on whether the target and the following probe were homophonic and click mouse buttons with their thumbs, with the left button for “different pronunciation” and the right button for “homophony.” The probes were high-frequency characters, including both single and compound characters. None of the single characters was a phonetic radical of any target character. For the compound-character probe, its phonetic radical was always different from that of the paired target. Half of the probes sounded the same with the targets, and the other half sounded different. The list of the target characters was presented twice so that the number of trials in each condition rose to 48. One target was paired up with different probes between the two presentations to reduce practice effects. 

### 2.4. EEG Recording and Analysis

The stimuli were programmed with the STIM software and randomly shown on a Lenovo monitor. Electroencephalographic (EEG) activity was recorded from a 64-channel NeuroScan version 4.3 system with a common vertex reference and rereferenced to the average of the left and right mastoids in the offline analysis. Vertical eye movements (VEOG) were recorded by a pair of electrodes placed on the supraorbit and infraorbit of the left eye, and horizontal eye movements (HEOG) were recorded by a pair of electrodes placed beside the outer canthus of both eyes. EEG signals were recorded and digitized at a bandpass filter of 0.05–100 Hz, with amplifying at a sample rate of 500 Hz. Resistances across all the electrodes were kept below 5 KΩ.

The acquired data were corrected for eye movements and blinks and segmented to epochs of 800 ms posttarget interval and 100 ms pretarget baseline. Epochs containing incorrect behavioral responses or with peak-to-peak differences larger than 100 *μ*v were rejected, and this led to a rejection rate of 8% over all trials, without statistical difference in the number of rejections between conditions (*P* > 0.1). The remaining epochs were filtered with a low-pass filter of 30 Hz (zero-phase shift mode, 12 dB) and averaged for each condition and participant. 

## 3. Results

The accuracy in homophonic judgement was higher than 90% for all participants.

**Figure 2:**
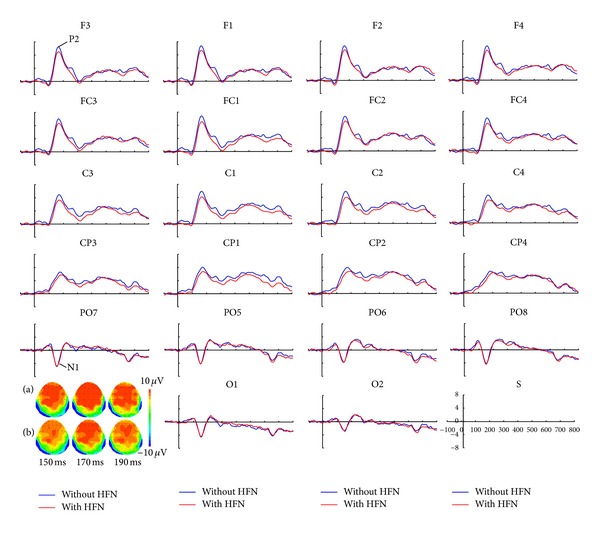
The ERP grand averages for the with-/without-HFN conditions in channels of F1-F2, F3-F4, FC1-FC2, FC3-FC4, C1-C2, C3-C4, CP1-CP2, CP3-CP4, PO5-PO6, PO7-PO8, O1-O2. The blue line indicates the without-HFN condition and the red line indicates the with-HFN condition. The topographic mapping was in the bottom left, with (a) for the without-HFN condition and (b) for the with-HFN condition.

Figure 2 displays the ERP grand averages time locked to the onset of target characters for neighborhood frequency comparison. As can be seen from the figure, the ERPs show a negative polarity peaked at around 150 ms over posterior regions and a positive polarity peaked at about 170 ms over frontocentral regions. They were identified as N1 and P2, respectively. The 40 ms time windows were selected centering the N1 and P2 peaks (N1: 130–170 ms; P2: 150–190 ms). According to topographic mapping in Figure 2, the N1 amplitude was output at channels of PO5-PO6, PO7-PO8, O1-O2, and the P2 amplitude was recorded at channels of frontal (F1-F2, F3-F4), frontocentral (FC1-FC2, FC3-FC4), central (C1-C2, C3-C4), and centroparietal (CP1-CP2, CP3-CP4) lobes. Mean ERP amplitudes from corresponding time windows were computed for each participant in both conditions. The N1 amplitude was submitted to a repeated-measure ANOVA with neighborhood frequency (NF, with/without HFNs) and laterality (left/right hemisphere). The P2 amplitude was submitted to a repeated-measure ANOVA, with three within-subject variables: neighborhood frequency (with-/without HFNs), laterality (left/right hemispheres), and lobe (frontal/frontocentral/central/centroparietal lobes). The Greenhouse-Geisser adjustment was used when sphericity assumption was violated [33]. 

The ANOVA on N1 amplitude showed no significant main effect or interaction (all *P*s > 0.1).

The ANOVA on P2 amplitude showed a significant main effect of neighborhood frequency (*F*(1, 12) = 12.88, *P* < 0.01), and ERP response to target characters was larger in without-HFN condition than in with-HFN condition; a significant main effect was also observed for lobe (*F*(1, 12) = 8.73, *P* < 0.01), and *post hoc* analysis showed the smallest amplitude in the centroparietal lobe but no differences across other three lobes; no significant effect was found for laterality (*F*(1, 12) < 1, *P* = 0.68) or any interactions (all *P*s > 0.1).

## 4. Discussion

In the present study, there was no effect of neighborhood frequency on bilateral posterior N1 amplitude; as to the P2 amplitude, naming Chinese characters without HFNs induced more positive ERPs than that with HFNs. The current results uncovered the time course of early influence of orthographic neighbors with higher frequency in Chinese-character naming.

The absence of neighborhood frequency effect in N1 amplitude revealed that higher-frequency neighbors did not affect visual/orthographic analysis of the target characters, since N1 is usually associated with the visual form processing of written words/characters [22, 26]. The finding was consistent with a previous study using English words, in which there was not HFN effect in the early time window before 180 ms [30]. Moreover, it was reported that the N1 originates predominately from the occipitotemporal regions [34]; thus the present result was identical with the nonsignificant HFN effect in the occipital areas reported by Li et al. [19]. 

The current amplitude difference between with- and without-HFN conditions in the P2 time-window indicated that higher-frequency neighbors exerted an influence on phonological processing of Chinese characters. Debruille also reported an HFN effect in English words with the ERPs response in the posttarget interval ranging from 180 ms to 240 ms [30]. These findings suggested the universal role of HFNs in phonological retrieval of target words/characters across language systems. However, the current HFN effect was greater P2 amplitude in without-HFN condition than that in with-HFN condition, while the neighborhood frequency effect in Debruille [30] was opposite. The difference might be ascribed to the language characteristics in phonological consistency. Considering the high phonological consistency in alphabetic writing systems, higher-frequency neighbors would provide support for the target phonology, which might correspond with increased brain activity of larger ERP amplitude. Whereas the phonological consistency was low in Chinese orthographic neighborhoods, different pronunciations of the HFNs would be more easily activated because of the lower threshold of activation for these neighbors and compete with the phonological activation of the target characters, resulting in the interference on phonological processing of Chinese characters, which may inhibit the relevant brain activity of P2 component. Additionally, the distribution of P2 effect was mainly located in the frontal and centroparietal areas. Neuroimaging studies reported that the left middle frontal gyrus was responsible for the addressed phonology, and the dorsal aspect of the left inferior parietal lobule was thought to be the region specific for phonological storage in Chinese; meanwhile, the right frontal regions were linked to phonological competition and inhibition [12, 19, 35, 36]. These results indicated that the higher-frequency neighbors might exhibit a disruptive effect in the lexical route of Chinese phonological access.

The present results were consistent with our prediction based on the analogy theory of word naming, revealing that orthographic neighbors with higher frequency might impact on target naming at the phonological level. Moreover, the finding also supported the inference in Li et al. [18]. Li et al. proposed that the orthographic neighborhood effect in Chinese-character naming may be generated in two phases: orthographic facilitation from visually similar neighbors and phonological inhibition of higher-frequency neighbors [18]. The current findings provided evidence for the phonological inhibition, suggesting negative influence of higher-frequency neighbors at the phonological retrieval in Chinese-character naming. 

## 5. Conclusions

The present study used ERPs to separately examine the effect of higher-frequency neighbors at the early orthographic and phonological stages. Results showed amplitude difference between with- and without-HFN conditions in P2 component but not in N1 component, suggesting a special role for the HFNs in addressed phonology of Chinese characters. The reduced amplitude of P2 for with-HFN characters might reveal the phonological inhibition from the higher-frequency neighbors due to their different phonological representations from the target characters.

## Figures and Tables

**Table 1:** Target characters and probes.

Without HFN			With HFN		
Target character	Probe 1	Probe 2	Target character	Probe 1	Probe 2
*诞*	*蛋*	*刺*	*掷*	*志*	*盲*
*懒*	*能*	*览*	*狄*	*笛*	*童*
*涤*	*串*	*迪*	*伐*	*罚*	*欧*
*肺*	*费*	*拼*	*晌*	*夜*	*赏*
*肆*	*四*	*抢*	*汁*	*知*	*梦*
*泄*	*贫*	*谢*	*坝*	*罢*	*灯*
*促*	*卖*	*醋*	*钥*	*亭*	*药*
*淀*	*赛*	*店*	*眨*	*肯*	*闸*
*徊*	*怀*	*粉*	*拆*	*送*	*钗*
*弥*	*迷*	*信*	*耕*	*庚*	*庞*
*啸*	*笑*	*宾*	*砍*	*琴*	*侃*
*诱*	*币*	*幼*	*靴*	*蚕*	*削*
*弦*	*嫌*	*穷*	*棍*	*票*	*森*
*谐*	*管*	*斜*	*锡*	*房*	*西*
*腮*	*塞*	*面*	*洗*	*喜*	*强*
*栓*	*闩*	*封*	*跌*	*爹*	*美*
*腾*	*疼*	*棒*	*妃*	*菲*	*飞*
*埃*	*船*	*直*	*绩*	*计*	*记*
*堪*	*热*	*刊*	*炉*	*图*	*层*
*愧*	*溃*	*奋*	*畔*	*盼*	*贵*
*钳*	*易*	*钱*	*恃*	*控*	*室*
*瞧*	*桥*	*乔*	*梯*	*剔*	*踢*
*撞*	*波*	*状*	*绰*	*缺*	*鲜*
*澄*	*星*	*成*	*坤*	*岗*	*昆*

HFN: higher-frequency neighbor.

**Table 2:** Stimulus characteristics in with- and without-HFN conditions.

	Without HFN	With HFN	*t*-test
	Mean (SD)	Mean (SD)	
Number of neighbors	8.00 (2.89)	7.92 (3.41)	ns
Frequency	11.00 (7.72)	10.39 (6.28)	ns
Stroke	10.33 (2.07)	9.46 (1.86)	ns
Consistency level	0.30 (0.13)	0.30 (0.17)	ns

HFN: higher-frequency neighbor. Frequency values are occurrences per million. ns: nonsignificant.
